# Barriers and enablers to vaccination in the ultra-orthodox Jewish population: a systematic review

**DOI:** 10.3389/fpubh.2023.1244368

**Published:** 2023-10-12

**Authors:** Avraham Jacobson, Sivan Spitzer, Yanay Gorelik, Michael Edelstein

**Affiliations:** Azrieli Faculty of Medicine, Bar Ilan University, Safed, Israel

**Keywords:** Measles, Polio, COVID-19, Religious, Minorities

## Abstract

**Background:**

The Jewish Ultra-Orthodox (UO) population is an under-vaccinated minority group that has been disproportionally affected by outbreaks of vaccine-preventable diseases (VPD) such as measles and polio. Underlying reasons remain poorly characterized. We aimed to identify vaccination barriers and enablers in this population.

**Methods:**

We systematically reviewed the literature (PROSPERO: CRD42021273001), searching Pub-med, Web of science, Medline, PsychNet and Scopus from 1995 to 2021 for quantitative and qualitative primary research in English. Studies published outside the date range, not including barriers or enablers, or that were non-primary research were excluded. We assessed included publications for quality and extracted relevant data based on the 5As taxonomy: access, awareness, affordability, acceptance and activation.

**Results:**

We included nine qualitative and seven quantitative studies from the 125 studies identified. Access barriers included scheduling difficulties, inconvenient opening hours, and logistical difficulties related to having multiple young children. Acceptance barriers included safety concerns. Insufficient knowledge about the importance of vaccine and timely vaccination and the perception of being shielded from infections because of seclusion from wider society were key awareness barriers. Competing priorities, such as work and housework, were the main affordability barriers. Mainstream religious leadership’s support for vaccination was an enabler, although recent studies suggest their influence on vaccination behavior is decreasing and influence of anti-vaccination messages is growing.

**Discussion:**

Barriers to vaccination among the UO were mainly logistical, with little religious framing. Safety and efficacy concerns were similar to those reported in the wider community. Decreasing influence of the traditionally pro-vaccine mainstream religious leadership and growing influence of anti-vaccination movements targeting the UO community are new phenomena that require close monitoring. Tailored interventions are required to protect the community and wider society against future VPD outbreaks.

**Systematic review registration:**

PROSPERO: CRD42021273001.

## Introduction

The Ultra-Orthodox (UO) are a distinct segment of Jewish society that stringently follows Jewish law and rabbinical leaders, opposing modern values to a greater or lesser degree (1, 2). The degree of obedience to rabbis and attitude toward modern values, have become more fluid in recent years, leading to more exposure to and interaction with general society (2). Yet, the UO remain a distinct population group within the wider societies in which they live.

The UO population is characterized by a very high birth rate [e.g., in Israel, 6.6 births per woman vs. 2.1 in the general population in 2020 (3)], and limited interaction with the wider society (1, 4). The largest UO population is in Israel, with sizeable communities in the United States, the United Kingdom and Belgium. These populations are largely inter-connected with a high volume of travel and communication between them, often leading to vaccine-preventable disease (VPD) outbreaks spreading from one community to others (5–7). UO Jews, while often considered a homogenous population group, are in reality a set of diverse sub-groups, each guided by their own religious leadership, and differing in their sources of influence, attitude to internal and external institutions and relationship with wider society. These differences may also impact on attitudes and behaviors toward vaccines and vaccination.

Previous studies have shown low vaccine coverage and delayed vaccination in UO communities (7, 8) as well as VPD outbreaks including: (a) Measles outbreaks in the United States in 2012–2013 and 2018–2019 (9, 10), Israel in 2003–2004, 2007–2008 and 2018–2019 (11–13); the United Kingdom in 2007–2008 and 2012–2013 (14) and Belgium in 2003–2004 (15); (b) Hepatitis A in the United Kingdom in 2010 (5); (c) polio cases in 2022 in Israel (16) and the United States (17, 18); (d) mumps outbreak in the United States in 2009–2010 (4); (e) pertussis outbreaks in the United States in 2014–2016 and in Israel in 2023 (19, 20). The COVID-19 pandemic has also disproportionally affected the UO population in Israel (21, 22) and the United Kingdom (23, 24). The disproportionate impact was partly related to socio-demographic and behavioral factors but also to low COVID-19 vaccine uptake (25, 26).

While low vaccine coverage is found among many minority groups (26–29), the determinants of vaccination are different in each minority group. Over the past decades, studies have been conducted on different aspects of vaccination in UO communities around the world, yet evidence regarding barriers and enablers to vaccination in the UO population and their evolution has not been systematically reviewed. The purpose of the present study is to systematically review the literature regarding barriers and enablers for vaccination in the UO population around the world. This review is also important for the development of effective interventions in the field. Developing effective tailored interventions requires that the vaccination determinants specific to each group is understood. This is consistent with the key objectives of the World Health Organization’s Immunization Agenda 2030 (IA2030) (30) that aims to achieve equitable vaccination for vulnerable populations.

## Methods

We systematically reviewed the peer-reviewed literature regarding barriers and enablers to vaccination in the UO population according to PRISMA guidelines (31) and registered with the International Prospective Register of Systematic Reviews (PROSPERO: CRD42021273001).

We considered several models categorizing drivers to vaccination for our review, including the 3c model, the 5c model and the 5a model (32). The 3c includes three factors for vaccine behavior: (a) Complacency refer to perceived risk of VPD (b) Convenience, referring to the ability of the individual to obtain vaccine such as accessibility and affordability and (c) Confidence, referring to trust in effectiveness and safety of vaccines and trust in the health systems. The 3c model was expanded to 5c by adding Calculation and Collective responsibility (33, 34). Calculation refers to information searching. People engaging in information searching tend to encounter a lot of misinformation on the internet and are more hesitant about vaccines. Collective responsibility refers to the willingness to make an effort and vaccinate myself to protect others in society.

There is considerable overlap in the concepts captured by the 3c, 5c and 5a theoretical models (33). We chose to describe the findings according to the 5As taxonomy framework (35). This framework focuses on pragmatic factors influencing vaccination that are non-socio-demographic and focuses on the division of the various barriers and enablers into a taxonomy that can be translated relatively easily into the development of intervention programs. The framework captures the determinants of vaccine uptake across five categories: (i) Access refers to the ability of individuals to be reached by, or to reach, recommended vaccines; (ii) affordability refers to the ability of individuals to afford vaccination, in terms of both financial and non-financial costs (e.g., time); (iii) awareness refers to the degree to which individuals have knowledge of the need for, and availability of, recommended vaccines and their objective benefits and risks; (iv) acceptance refers to degree to which individuals accept, question or refuse vaccination; and (v) activation refers to the degree to which individuals are nudged toward vaccination uptake (35). In addition, the 5A framework was previously used in another systematic review that dealt with routine vaccination among minority populations in high income countries (27). Its pragmatic approach, previous use in systematic review and the authors familiarity with the model influenced our choice.

### Inclusion and exclusion criteria

The inclusion and exclusion criteria were developed using a PICOS (36) framework (Table 1). We included quantitative and qualitative primary research studies that contained data on barriers or enablers to uptake of any vaccine (including COVID-19) in UO populations, published Jan 1, 1995–November 21, 2021 in English. Studies involving Health Care Professionals (HCPs) working with UO populations were also included to capture provider-level and system-level perspectives pertaining to our primary outcome. We excluded non-primary research articles such as reviews, commentaries or opinion pieces.

**Table 1:** Inclusion and exclusion criteria, using PICOS framework.

	Inclusion criteria	Exclusion criteria
Population	Adult, adolescent and child who belong to the ultra-orthodox Jewish population; HCP’s (doctors, nurses, health care assistances, etc.) who work with or have worked with the ultra-orthodox Jewish population	Population are not belong to the ultra-orthodox Jewish population or are not HCP’s (doctors, nurses, health care assistances, etc.) who work with or have worked with the ultra-orthodox Jewish population
Intervention	Vaccination	Not vaccination
Control	No control was selected for this review	Not applicable
Outcomes	Barriers and enablers to vaccine uptake in Ultra-orthodox Jewish populations.	Not barriers or enablers to vaccine uptake.
Study design	Primary research	Non-original research articles (e.g., reviews, commentaries, editorials, case reports, and guidelines on vaccination)

### Search strategy

**Figure 1:**
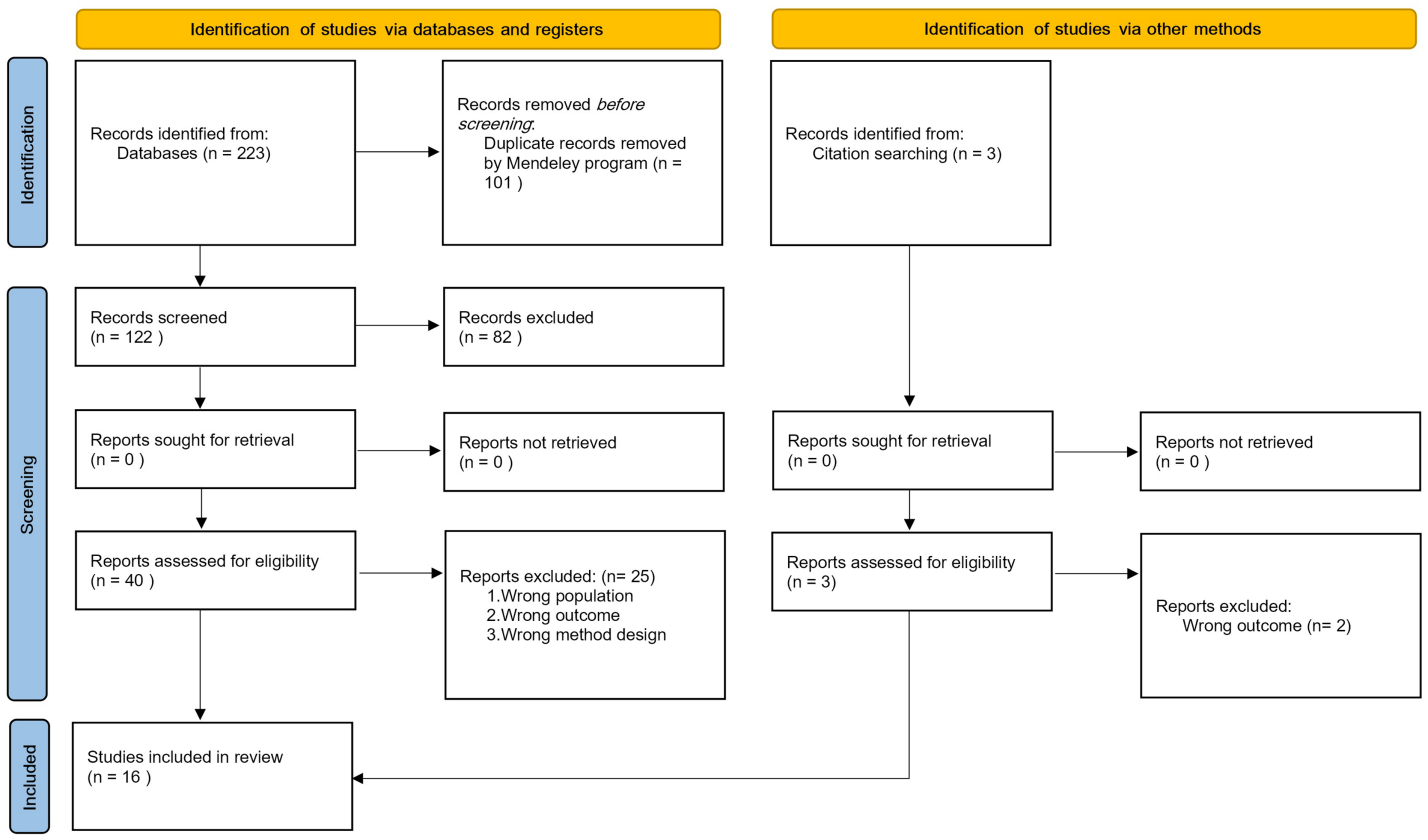
PRISMA flow diagram for strategy of identification, screening and inclusion of studies reporting barriers to and enablers of vaccination among the ultra-orthodox Jewish population, from January 1995 to November 2021.

We searched Pub-med, Medline, Web of Science, PsycNet and Scopus databases, combining free-text terms and subject headings relating to (ultra-orthodox Jewish) AND (vaccination; see Appendix A, B for search terms). Bibliographies of included studies were also hand searched for additional relevant references. Records were imported into Mendeley, and duplicates deleted. Title and abstract screening and full-text screening were independently carried out by two reviewers (AJ and YG) using Rayyan QCRI (37). The selection process is shown in Figure 1.

### Data extraction

Data were independently extracted by two reviewers (AJ and YG) and included location and year of study, study design, vaccine(s), vaccination type, barriers and enablers. Discrepancies at any stage were resolved through discussion with a third reviewer (ME) until a consensus was reached.

### Quality assessment

Quality assessment of the included studies was carried out independently by two reviewers (AJ and YG) using the Joanna Briggs Institute (JBI) critical appraisal tool which provides a separate checklist and rankings for qualitative and quantitative studies (38) grouping them into high (score of 80+), medium (score 50–79) and low (score < 50) quality. Studies were not excluded from this study based on quality assessment in order to increase transparency.

### Data synthesis and analysis

Extracted data were tabulated and results presented as reported in the studies. All data were synthesized narratively. Qualitative and quantitative data were first analyzed thematically to identify factors influencing uptake, then categorized using the 5As taxonomy (35), and further classified by emergent subthemes.

## Results

We identified 223 articles from the searched databases and removed 101 duplicate records. Three articles were added following hand searching references. Eighty-two records were excluded based on the title or abstract. The articles were excluded based on an out of scope population (e.g., focusing on a population other than ultra-Orthodox), out of scope outcomes (e.g., articles describing outbreaks without information on barriers or enablers), or inappropriate methods (e.g., experts’ opinion without evidence). We screened 43 full-text articles for eligibility of which 16 were included (Figure 1), with a combined sample size of 1334 UO parents. We could not quantify the number of HCPs interviewed, because not all studies reported their exact number. Most studies reported on childhood routine vaccinations, one study on COVID-19, one on influenza and one examined vaccination in general. Studies were conducted in Israel, United States, United Kingdom and Belgium. Designs included cross-sectional (*n* = 5), cohort (*n* = 1) outbreak report (*n* = 1) and qualitative (*n* = 9). Detailed characteristics of included studies are shown in Appendix C.

Access and acceptance were the most common themes, with awareness, affordability, and activation less reported. Unique subthemes relating to barriers and enablers to uptake were defined and are summarized in Table 2.

**Table 2:** What are the barriers to and enablers of vaccine uptake in UO population?

	Access	Acceptance	Awareness	Affordability	Activation	Other
Barriers	∙ Logistic difficulties related to large households with young children. For example, the mothers will find it difficult to actually get to the clinic when she has several other young children at home (8, 13, 15, 39–43) ∙ Challenges in getting an appointment due to availability and accessibility issues, the complicated appointment system, and the inconvenient hours of operation (39–41, 44) ∙ Negative experiences, such as lack of child-friendly facilities, overload, and sometimes stressful environment (8, 40) ∙ Unsympathetic treatment by practice staff (39, 41) ∙ Time pressure on healthcare workers does not leave them free time to address parents’ concerns (39, 40)	∙ Worries about vaccine safety and side effects (7, 15, 39–41, 45, 46) ∙ Mistrust in the medical and sometimes also religious systems (40–42, 45–47) ∙ Pain and discomfort associated with childhood vaccinations (8, 48) ∙ Religious factors: e.g., religious fatalism, religious belief against vaccination (42, 49) ∙ Concern over new vaccines (pneumococcal and rotavirus vaccine) or those not included in the program (influenza vaccine in Israel) (8) ∙ Perception that children are receiving too many vaccines too early in life (7) ∙ Vaccination not considered a social norm (40, 49)	∙ Lack of information about diseases or the need for vaccination (8, 40, 49) ∙ Low perception of risk of disease or important of vaccination (8, 39, 42, 49) ∙ Lack of information or misperception about the importance of timeliness in vaccination (8, 39, 40) ∙ Insufficient knowledge about childhood vaccines: e.g., schedule, types (8) ∙ Personal health stewardship: e.g., knowing personal vaccination history (8, 40) ∙ Lack of information or misperception of possible side effects (15, 39) ∙ Reliance on community rumors rather than official information (48, 49) ∙ Perception of protection from vaccine preventable diseases as a result of isolation from wider society (49)	∙ Competing priorities in large family size with primary breadwinner mothers, which leads to dealing with a variety of personal and professional responsibilities, and the difficulty of prioritizing vaccines (8, 15, 39–42) ∙ Indirect costs, such as loss time from work (41)	∙ Timing of giving the information to mothers about vaccines is problematic: many mothers did not remember that they received information about vaccines (8) ∙ Lack of vaccination in school, in Antwerp, Belgium’s (15) ∙ Lack of HPV vaccination in UO schools (50)	∙ Specific targeting of the UO community by anti-vaccine organizations (41, 47)
Enablers	∙ Convenient location of immunization Clinics (44) ∙ Most mothers expressed high confidence in the nurses’ professional expertise (8, 44)	∙ Support for vaccination among most religious leaders (40, 41, 45, 46) ∙ Most parents hold positive attitudes in the medical system (8, 44)	∙ Oral explanation from medical staff regarding vaccines (44) ∙ Most mothers recognized the risk of not being vaccinated, and of the safety of vaccines (44)	∙ Cost offsetting: free vaccination (42)		∙ Existence of effective intervention programs tailored to the UO population (7, 13, 40, 43, 51)

### Access to vaccination

Logistic difficulties related to large households with young children were commonly reported as barriers to uptake (8, 15, 39–42). For example, getting to the clinic when several other young children are at home was a challenge commonly reported by mothers. System-level barriers included difficulties in making vaccination appointments due to unclear or inconvenient operating hours, and a complicated appointment system (39–41, 44).

Although these systemic barriers affect the entire population, they are particularly detrimental to populations who initially have difficulty reaching out. Other barriers included negative experiences in busy, overcrowded and sometimes stressful clinics (8, 40), lack of child-friendly facilities (40) and unsympathetic treatment by practice staff (39, 41). Healthcare providers reported being under increasing pressure and not always having the time and availability to respond to the mothers’ demands (39, 40).

Conversely, clinics located in convenient and accessible locations (44) and high confidence in the nurses’ professional expertise (8) were perceived as enablers to vaccination.

### Acceptance of vaccination

Several studies noted safety and side-effects concerns as barriers to vaccination uptake (7, 15, 39, 40, 42, 45, 46). Worries about “overloading” the child’s immune-system with multiple or combined vaccines too early in life, concern for the child’s pain (48) and fear of death, paralysis or autism (for MMR vaccines), were highlighted by some UO groups. Studies noted greater concern for newer vaccines (e.g., pneumococcal and rotavirus vaccines) or those that are perceived as outside the program (e.g., influenza vaccine in Israel) (8). Safety concerns stemmed from a decrease in trust in the medical establishment in general (40–42, 45–47) and ranged from hesitancy (7, 8, 39, 40) to complete opposition to vaccines (41, 45–47). Sources of influence in these cases also varied, ranging from rumor-based (8, 39) through misinformation by community physicians (15) to content provided by anti-vaccination movements (41, 45–47). In some cases, those reporting complete opposition to vaccinations also described a loss of trust in the rabbinical establishment (41, 45, 46).

Another barrier was low perceived importance of vaccination and risk of vaccine-preventable disease (8, 39, 40, 46, 49). A lack of familiarity with the diseases’ potential consequences, a fatalistic religious worldview and a sense of protection based on the relative isolation of the community from the rest of the population all contributed (49). Several studies pointed to broad acceptance of vaccination among UO populations but low awareness of the importance of receiving vaccinations on time, leading to delaying vaccination until a later age (8, 41, 49).

The main enabler related to acceptance one was the pro-vaccination stance of rabbinic leadership in UO groups. The cooperation between religious and health authorities around interventions to encourage vaccinations illustrate this stance (13, 40). The finding that even parents who oppose vaccinations are aware that they are acting contrary to the opinion of the rabbis (41, 46) provide further evidence for the support of religious leadership. In addition, although UO trust in the medical establishment may be declining, large segments of the UO population still holds a positive attitude toward the medical system, which facilitates vaccination acceptance (8, 44).

### Awareness of need for vaccination

Knowledge barriers in UO populations include lack of knowledge about VPD, the need for vaccination (8, 40, 49), what childhood vaccines are in the schedule and the importance of adhering to it (8, 39, 40). Parental lack of recall of their children’s vaccination history, compounded by a high number of children, led to confusion about what vaccines are required for which child (8, 40). Lack of information or misinformation on possible side effects (15, 39, 41, 45–47) and reliance on intra-community rumors instead of formal and reliable sources of information also contributed (49).

In terms of awareness enablers, most mothers recognized the risk of being unvaccinated and thought vaccines were safe (8, 44). Received an oral explanation about vaccines from the medical staff was also enabling (44).

### Affordability of vaccination

All countries where UO communities live offered free routine vaccination but indirect costs, such a loss of time from work, existed (41). This barrier was particularly relevant in Israel where UO mothers are usually the main household earner. Competing priorities, such as childcare and household chores, were non-financial barriers to vaccination among UO parents, including those who were positive about vaccination or intended to vaccinate their children. An ordinary UO household has several children of vaccination age, and in many cases the mother works and is also responsible for the children’s upbringing, including vaccinations. This societal reality means UO mothers must simultaneously manage many personal and professional responsibilities, making prioritizing vaccination a challenge (8, 15, 39–42).

### Activation and nudging toward vaccination

Specific circumstances external to healthcare systems can affect activation of vaccination in the UO population. One Belgian study described how the school health service that delivers certain vaccines in schools did not serve the private schools enrolling the vast majority of UO children, making them reliant on pediatricians and GPs for vaccination (15). Because the vast majority of children in this community attend schools catering exclusively to the community, the UO schooling system can control to some extent what vaccines to promote. A UK study showed lower vaccine coverage for HPV but not meningococcal ACWY disease vaccines (both school-delivered) in Jewish schools compared to other schools (50). Another barrier is the fact that mothers receive information about their child’s vaccinations only when they are already at the clinic and not before, which makes it difficult for them to prepare for further vaccination (8).

### Other factors unique to the ultra-orthodox population

Recent studies show that anti-vaccination groups specifically target the UO community with misinformation, leading to pockets of resistance to vaccination within the UO population (41, 46, 47). One study documented in 2019 in Israel, two anti-vaccination conferences led by anti-vaccination activists from the United States and Europe, specifically targeting the UO community. They included lectures about the right to refuse vaccination, using imagery and messaging to which the community would be specifically responsive, such as the Holocaust (47).

Because UO communities, especially those outside of Israel and the US, are extremely tight-knit and quite small, individual healthcare workers can disproportionately influence the entire community’s susceptibility to VPD. In Antwerp, Belgium, two doctors providing healthcare to a high proportion of the UO community advised parents against vaccinating their children (15), making the entire community vulnerable.

Positively, a number of interventions specifically designed to increase vaccine uptake in UO communities were shown to be effective, especially following outbreaks (7, 13, 40, 43, 51). These included collaborative campaigns with UO religious leaders and stakeholders and improved vaccine accessibility. For example, following the 2018 Jerusalem measles outbreak, a collaborative campaign with religious leaders along with the extension of maternal child health clinics (MCHC) hours from 8.00 to 20.00 (43) was associated with an increase in MMR uptake from 76.3 to 96.1% within 30 weeks in intervention neighborhoods (43).

## Discussion

Our review shows that among the five categories considered (access, acceptance, awareness, affordability and activation), access barriers were of key importance and included logistic difficulties related to large households with young children, and service barriers such as inconvenient opening hours and a complicated appointment system for key vaccines including MMR, DPT, influenza, polio and COVID-19 vaccines. Acceptance barriers were also reported and included concerns about safety and side-effect and worries about “overloading” the immune-system. In terms of awareness, barriers included a lack of knowledge about the schedule and need for timely vaccination, and a sense of protection stemming from social isolation from wider society (49). Even in the context of freely available vaccines, competing priorities such as work and housework commitments constituted indirect affordability barriers. In terms of activation, schools and local healthcare providers in some instances limited access and perceived need for vaccines. This was particularly the case for HPV vaccination where the feeling of not being at risk because of conservative norms in sexual behavior in the community was commonly expressed (52).

Our review also highlights several enablers, including efforts to make vaccination services accessible, trust in healthcare professionals and support from mainstream rabbinic leadership. Although rabbinical endorsement was essential for the COVID-19 vaccination campaign in Israel (53), there are indications the community is becoming less obedient to religious authority. Low vaccine COVID-19 coverage in this population group, despite rabbinical endorsement at the highest level, to outright defiance of rabbinical rulings on the issue has been recorded (41). The studies provided encouraging evidence of effective interventions in this populations. However, successful interventions were implemented reactively following outbreaks, so it remains unclear whether they would also succeed outside of a crisis context. In addition, tailored interventions to address low vaccine coverage in underserved populations are generally funded as “projects” and often suffer from a lack of sustainable funding (54), with the situation reverting as funding for the intervention ends.

The access barriers, especially difficulty in finding time to get vaccinated among large families with young children, were the most consistently mentioned barriers, over time as well as across different countries. These findings were consistent with studies showing how the multiple burden that UO mothers experience makes it difficult for them to find time and energy to devote to tasks they perceive as less urgent, such as preventive and promotive medicine (55, 56). One study, for example, described the tension that UO mothers experience between the centrality of motherhood in the lives of UO women, vs. the difficulty of being successful and meeting all the responsibilities that come with having a family with many young children (55). The predominant assignment of responsibility for child vaccination to mothers underscores the persistence of gender role inequality, wherein the primary duty for child-rearing and safeguarding their health is placed on mothers. These observations align with broader societal trends indicating that, even within the general population, the primary responsibility for vaccinating children is often borne by mothers (46, 57). This pattern is further reflected in the notable representation of mothers among activists within anti-vaccine movements (46, 57). Within the UO community, this responsibility is compounded by the additional burden of household provision, which frequently falls upon the household head. This stress, along with a lack of sufficient knowledge, affects mothers’ ability to meet the needs of their children in various aspects of general health beyond vaccination, such as nutrition, physical activity and sleep (55). The low vaccine-related knowledge we identified fits within the context of low levels of generic scientific education in UO schools where girls typically study science until age 15 and boys rarely study science beyond ages 11–12, since it is considered unnecessary for a religious scholar (58). Science literacy scores among UO girls in PISA tests (Program for International Student Assessment, by the OECD) in Israel in 2018 were low compared to the rest of the population (59). The interplay between scientific knowledge and health decisions among the UO is complex, as demonstrated by adherence to COVID-19 restrictions (60). An additional study highlighted the gap between the low level of formal scientific education within the UO education system and the confidence of UO individuals in the community’s medical knowledge, especially among informal health experts (61). These findings highlight the need for further research on how scientific knowledge in the UO community affects vaccine compliance.

Our review also highlighted a mismatch between the perceived sense of security from VPD in some UO communities (49) and the epidemiological reality. The high number of VPD outbreaks that have affected UO populations in the past two decades, including polio (16), measles (7), mumps (4) and hepatitis A (5), have made it arguably among the most VPD-outbreak-prone group among minorities in high-income countries (62). Yet, the extent to which the UO are aware that VPD outbreak incidence in their communities is likely higher than in almost any other group is not clear. Beyond under-immunization in the UO population, are other risk factors associated with the UO may contribute to a higher risk of VPD transmission in this population, some of which mentioned in the literature. These include high population density in crowded dwellings (13); large and crowded gathering, including weddings with guests from several countries attending (13); crowded mass gathering events such as the annual religious pilgrimage to the town of Uman, Ukraine that propagated a measles outbreak in 2019 (10, 63); and prolonged hours of spent face to face in close proximity when learning religious texts (4). Further research should investigate the community’s awareness of increased risk and whether increasing the community’s awareness of these risk factors can be used as an enabler in future interventions targeting under vaccination.

The studies included in this review consistently show that the relatively low vaccine coverage in UO populations does not stem from theological opposition to vaccination. The “Gedoley hador”: the most respected and influential rabbis support vaccination (64) and their endorsement were an important part of intervention programs (13, 40). Mothers who considered themselves anti-vaccination were aware that they were acting contrary to rabbinical opinion, exemplifying awareness that the prevailing religious position supports vaccinations (46). HPV may be a notable exception, not because of theological opposition *per se* but because of the perception that an orthodox lifestyle, where early marriage and a single lifetime sexual partner is the norm, eliminates the risk of HPV infection (52). Indeed, despite no religious objections to HPV vaccination in principle, both parents and community leaders report HPV as being unnecessary since multiple sexual partners is not part of the UO lifestyle (52). Data on sexual behavior among young UO is sparse and it is hard to determine the extent to which this position reflects reality or whether accepting HPV vaccination in school would be perceived as an admission that pre-marital sex does indeed occur. Compared with secular Jewish women, UO women have a much lower proportion of abnormal cervical smear tests, suggesting differential risk (65). Either way, attempts to introduce HPV vaccination in UO schools in Israel have largely been resisted (64). While we have anecdotally identified fringe antivaccination voices in the rabbinical world (66, 67), existing research refers mainly to mainstream rabbinical voices. The influence of these fringe anti-vaccination rabbis may become more relevant in a post-COVID world where vaccination has become a polarizing issue and the issue warrants further investigation.

Studies published until the late 2010s assumed the UO community fully obeyed rabbinical authority (1, 4) with a consensus that interventions targeting this community, on vaccines or otherwise, relied on engaging religious authorities (40, 43). Studies published in the early 2020s suggest the emergence of pockets of vaccine resistance despite mainstream rabbinical opinion (45–47). Individual decision-making contrary to rabbinical opinion is a new phenomenon that inscribing itself in a broader change process within the UO community, including factors such as the community’s exponential growth (2, 68) exposure to the Internet (69), more UO individuals attending higher education (70, 71), NGOs promoting exposure of sexual abuse in the community in the spirit of the me-too movement (72), and the growth of civil leadership alongside rabbinical leadership (72). These changes are diversifying the UO population and challenging centralized authority. In the context of vaccination, mothers exposed to the Internet may balance rabbinical opinion with online information to make a decision. Because of low science and IT literacy in this population (60, 73), young UO parents may struggle to evaluate the reliability and legitimacy of information they encounter online, with direct implications for vaccine confidence.

The correlation between a relative decrease in conservatism, increase in individual decision-making, decrease in the influence of central religious authority and an increase in opposition to vaccination seen in the UO population differs from other religious groups. They are almost the opposite of dynamics at play among the Orthodox Protestant Christian groups (OP) which vaccine coverage was low due to religious objection (62, 74) but increased as younger community members became less obedient to religious leadership, less conservative and more exposed to general culture (75). This difference illustrates that there is no simple and direct relationship between religion, conservative views and attitudes toward vaccines, as the relationship between religion and vaccination is closely influenced by the cultural and social context. Each conservative or religious community should be examined separately: findings on vaccination behavior from one religious/conservative community may not apply to another.

UO exposure to anti-vaccination movements is another new phenomenon. Part of the exposure is through online content on the Internet or social media (45, 46) but there is also an emerging phenomenon of activists from anti-vaccination movements who work to actively influence the ultra-orthodox public (47). Rooted in Christian or secular ideology, anti-vaccination movements’ campaigns have specifically target UO communities in Israel, using a religious framing and cultural references specifically tailored to this population, including analogies to the Holocaust (47). This new phenomenon of a Christian anti-vaccination movements from the United States influencing the UO community in Israel is a clear product of accelerated globalization processes, which enable the rapid migration of ideas and their conversion from a religious language to a “secular” language and vice versa (76). These findings are in line with other studies that have shown how globalization allows anti-vaccination ideologies to spread and penetrate distant and different cultures (77, 78).

The findings of the current review show that future intervention programs will have to be tailored to the community and address evolving barriers to vaccination. Rabbinical endorsement is important but no longer likely to be sufficient. Interventions that are likely to be successful should consider logistical components such as adapted hours and facilities (40), as well as components that will address gaps in trust between members of the UO community and public health services. One of the examples of an intervention method that can help is collaboration with welfare UO organizations, that the community members trust and that can provide services in designated clinics within the community (79).

A key limitation of our review is that because of the low number of relevant studies on the topic, we could not meaningfully compare drivers to vaccination in different scenarios such as routine vaccination in health centers, school vaccination, routine adult vaccination, and emergency vaccination as an outbreak response measure. In practice, barriers for each of these scenarios are likely to be different. The availability of several valid theoretical frameworks available complicated our choice of analytical approach. The key factors influencing our choice included familiarity with the framework, previous use of the framework on similar topics, the opportunity for easy translation to practical recommendations and the inclusion of dimensions beyond vaccine hesitancy. Ultimately, because of the considerable overlap between the models, we feel that the use of a different model would have identified the same barriers albeit presented differently.

To conclude, our study suggests that vaccination decision making processes among the ultra-orthodox communities are becoming more diverse, complex and individual, highlighting heterogeneity and change within the community. While logistical and access barriers remain central, vaccine hesitancy, defined as a state of indecisiveness regarding a vaccination decision (80), has begun to emerge in recent years, likely as a result of societal changes within the community leading to exposure to anti-vaccination material, compounded by a lack of skills to critically appraise this new information. Our findings show that interventions using an exclusively religious framing are unlikely to be effective and highlight the need to develop and sustainably implement tailor-made interventions that match actual barriers, for specific UO sub-communities in order to increase vaccine coverage in these populations.

## Data availability statement

The original contributions presented in the study are included in the article/Supplementary material, further inquiries can be directed to the corresponding author.

## Author contributions

ME initially conceived this review. AJ and YG independently conducted and verified the searches, screening, data extraction, and analysis. AJ wrote a first draft of the paper with guidance and editing from ME and SS. All authors discussed the findings and contributed to the review and editing of the final manuscript. All authors had full access to the full data in the study and accept responsibility for the decision to submit for publication. All authors contributed to the article and approved the submitted version.
