# Supplementary material for: Barriers and enablers to vaccination in the ultra-orthodox Jewish population: a systematic review
Source: Front Public Health. 2023 Oct 12;11:1244368. doi: 10.3389/fpubh.2023.1244368 (PMC10602685; doi:10.3389/fpubh.2023.1244368)
Supplement: Supplementary file 1 [file Table_1.DOCX]

Supplementary Material

**Appendix A: keywords (consistent across all databases).**

| **Theme** | **Keywords** |
| --- | --- |
| Ultra-orthodox Jewish | Orthodox Jews OR Ultraorthodox Jews OR Ultra Orthodox Jews OR Ultra-orthodox Jews OR Orthodox Jewish OR Ultraorthodox Jewish OR Ultra Orthodox Jewish OR Ultra-orthodox Jewish OR Charedim OR Charedi OR Haredim OR Haredi |
| Vaccination | Vaccination OR Vaccine OR Immunization OR Immunisation OR Inoculation |

**Appendix B: Example search terms: MeSH terms (varied for each database; example below used for Pub-Med).**

| **Theme** | **Keywords** |
| --- | --- |
| Ultra-orthodox Jewish | Orthodox Jews OR Ultraorthodox Jews OR Ultra Orthodox Jews OR Ultra-orthodox Jews OR Orthodox Jewish OR Ultraorthodox Jewish OR Ultra Orthodox Jewish OR Ultra-orthodox Jewish OR Charedim OR Charedi OR Haredim OR Haredi |
| Vaccination | vaccination (Mesh) OR immunization (Mesh) |

***Appendix C*: Characteristics of included studies reporting barriers to and enablers of vaccination among the ultra-orthodox Jewish population, from January 1995 to November 2021, including study quality (based on JBI critical appraisal tools and then organized into L (Low: < 49%) M (Medium: 50-79%) and H: (High: <79%) (n=16)**

| **Study author(s)** | **Year(s) of study** | **Location** | **Study design** | **Method** | **Vaccine** | **Sample size** | **Study population** | **Factors identified** | **Quality** | |
| --- | --- | --- | --- | --- | --- | --- | --- | --- | --- | --- |
| Elran et al.(1) | 2014-2016 | Israel | Cross-sectional | Survey | Childhood vaccination | 378 | Parents | Awareness, acceptance | 87.5% | H |
| Henderson et al.(2) | 2003 | North East London, UK | Quantitative | Semi-structured interviews | BCG, MMR, DTV | Mothers: 25  Health care workers: 10 | Mothers and local health care workers. | Awareness, acceptance | 80% | H |
| Kasstan B.(3) | 2019-2020 | Jerusalem, Israel | Qualitative | Semi structured interviews and ethnographic research | Vaccines in general | 22 interviews. Ethnographies of 2 anti-vaccine events | Parents who refused vaccinations | Other | 80% | H |
| Kasstan B.(4) | 2014-2015, 2018-2019, 2020-2021 | Manchester, UK. Jerusalem, Israel | Qualitative | Semi structured interviews, text analysis and ethnographic research | Covid-19 and routine vaccines | 66 interviews, Text analysis of health information and rabbinic texts | Parents, formal and informal healthcare practitioners, and religious leaders. | Access, acceptance,  affordability,  other | 80% | H |
| Keshet et al.(5) | 2019 | Israel | Qualitative | In depth interviews | Childhood vaccination | 10 | Mothers who declared that they do not vaccinate their children | Acceptance | 90% | H |
| Lernout et al.(6) | 2007-2008 | Antwerpian, Belgium | Cross-sectional | Face to face and telephone survey | MMR | 69 | Measles patients or parents of measles patients | Access, awareness, acceptance, activation,  other | Not applicable | |
| Letley et al.(7) | 2015 | Hackney, London, UK | Qualitative | Semi-structured and open interviews | Childhood vaccination | 136 | Parents and community stakeholders | Access, awareness, acceptance | 80% | H |
| Loewenthal et al.(8) | Not applicable | Stamford Hill, London, UK | Qualitative | Semi-structured interviews and focus group | DTP,  MMR,  TB | Interviews:10  Focus group: not applicable | Mothers, GP's, health visitors and practice manager | Access, awareness, acceptance | Not applicable | |
| Mushen et al.(9) | Not applicable | Bnei- Brak city and Jerusalem district, Israel | Cross sectional | Telephone survey | HBV,  DT,  P,  HIB,  IPV,  MMR, HAV | 430 | Parents | Access, awareness, acceptance | 87.5% | H |
| Popper Giveon et al(10) | 2019 | Israel | Qualitative | In depth interviews | Childhood vaccination | 10 | Mothers who declared that they do not vaccinate their children | Acceptance | 100% | H |
| Simhi et al.(11) | 2009 | Center and south of Israel | Cross sectional | Survey | HBV,  IPV,  DTaP,  Hib,  PCV, MMR, VAR. | 85 | Mothers | Access, awareness, acceptance | 87.5% | H |
| Stein Zamir et al.(12) | Not applicable | Jerusalem district, Israel | Qualitative | Semi-structured interviews and focus group | MMR, DTaP4, Influenza, pneumococcal, rotavirus | Focus group: 45,  Interviews: 42 | Mothers | Access, awareness,  Acceptance, activation | 90% | H |
| Stein Zamir et al.(13) | 2018 | Jerusalem, Israel | Cohort | Data analysis of vaccination coverage rate | MMR | Not applicable | Children aged 1-14 years | Access, awareness | 62.5% | M |
| Stein Zamir et al.(14) | 2003-2004 | Jerusalem, Israel | Outbreak report | Data analysis of vaccination coverage rate | MMR | Not applicable | Toddlers aged 1–5 years | Access, awareness | 87.5% | H |
| Yamin et al.(15) | 2011 | Israel | Cross sectional | Survey | Influenza vaccine | 72 | Adults above 18 years | Access, awareness | 87.5% | H |
| Zucker et al.(16) | 2010 | Williamsburg, Brooklyn, New York, USA | Qualitative | Focus group | Childhood vaccination | Not applicable | Mothers | Awareness,  acceptance | Not applicable | |

**References**

1. Elran B, Yaari S, Glazer Y, Honovich M, Grotto I, Anis E. Parents’ perceptions of childhood immunization in Israel: Information and concerns. *Vaccine* (2018) 36:8062–8068. doi: 10.1016/j.vaccine.2018.10.078

2. Henderson L, Millett C, Thorogood N. Perceptions of childhood immunization in a minority community: Qualitative study. *J R Soc Med* (2008) 101:244–251. doi: 10.1258/jrsm.2008.070363

3. Kasstan B. “A Free People, Controlled Only by God”: Circulating and Converting Criticism of Vaccination in Jerusalem. *Cult Med Psychiatry* (2022) 46:277–296. doi: 10.1007/s11013-020-09705-2

4. Kasstan B. “If a rabbi did say ‘you have to vaccinate,’ we wouldn’t”: unveiling the secular logics of religious exemption and opposition to vaccination. *Soc Sci Med* (2021) 280:114052. doi: 10.1016/j.socscimed.2021.114052

5. Keshet Y, Popper-Giveon A. “I Took the Trouble to Make Inquiries, So I Refuse to Accept Your Instructions”: Religious Authority and Vaccine Hesitancy Among Ultra-Orthodox Jewish Mothers in Israel. *J Relig Health* (2021) 60:1992–2006. doi: 10.1007/s10943-020-01122-4

6. Lernout T, Kissling E, Hutse V, De Schrijver K, Top G. An outbreak of measles in orthodox Jewish communities in Antwerp, Belgium, 2007-2008: different reasons for accumalation of susceptibles. *Eurosurveillance* (2009) 14:15–18. doi: 10.2807/ese.14.02.19087-en

7. Letley L, Rew V, Ahmed R, Habersaat KB, Paterson P, Chantler T, Saavedra-Campos M, Butler R. Tailoring immunisation programmes: Using behavioural insights to identify barriers and enablers to childhood immunisations in a Jewish community in London, UK. *Vaccine* (2018) 36:4687–4692. doi: 10.1016/j.vaccine.2018.06.028

8. Loewenthal KM, Bradley C. Immunization uptake and doctors’ perceptions of uptake in a minority group: Implications for interventions. *Psychol Heal Med* (1996) 1:223–230. doi: 10.1080/13548509608400020

9. Muhsen K, Abed El-Hai R, Amit-Aharon A, Nehama H, Gondia M, Davidovitch N, Goren S, Cohen D. Risk factors of underutilization of childhood immunizations in ultraorthodox Jewish communities in Israel despite high access to health care services. *Vaccine* (2012) 30:2109–2115. doi: 10.1016/j.vaccine.2012.01.044

10. Popper-Giveon A, Keshet Y. Non-Vaccination Stage Model (NVST): The decision-making process among Israeli ultra-orthodox Jewish parents. *Heal (United Kingdom)* (2022) 26:777–792. doi: 10.1177/1363459320988884

11. Simhi M, Shraga Y, Sarid O. Vaccination of infants and health beliefs of ultra-orthodox mothers. *J Vaccines Vaccin* (2014) 5:2–8. doi: 10.4172/2157-7560.1000213

12. Stein Zamir C, Israeli A. Knowledge, Attitudes and Perceptions About Routine Childhood Vaccinations Among Jewish Ultra-Orthodox Mothers Residing in Communities with Low Vaccination Coverage in the Jerusalem District. *Matern Child Health J* (2017) 21:1010–1017. doi: 10.1007/s10995-017-2272-5

13. Stein-Zamir C, Abramson N, Edelstein N, Shoob H, Zentner G, Zimmerman DR. Community-oriented epidemic preparedness and response to the Jerusalem 2018–2019 measles epidemic. *Am J Public Health* (2019) 109:1714–1716. doi: 10.2105/AJPH.2019.305343

14. Stein-Zamir C, Zentner G, Abramson N, Shoob H, Aboudy Y, Shulman L, Mendelson E. Measles outbreaks affecting children in Jewish ultra-orthodox communities in Jerusalem. *Epidemiol Infect* (2008) 136:207–214. doi: 10.1017/S095026880700845X

15. Yamin D, Gavious A, Davidovitch N, Pliskin J, S. Role of intervention programs to increase influenza vaccination in Israel. *Isr J Health Policy Res* (2014) 3:1–7. doi: 10.1186/2045-4015-3-13

16. Zucker JR, Rosen JB, Iwamoto M, Arciuolo RJ, Langdon-Embry M, Vora NM, Rakeman JL, Isaac BM, Jean A, Asfaw M, et al. Consequences of Undervaccination — Measles Outbreak, New York City, 2018–2019. *N Engl J Med* (2020) 382:1009–1017. doi: 10.1056/nejmoa1912514

**
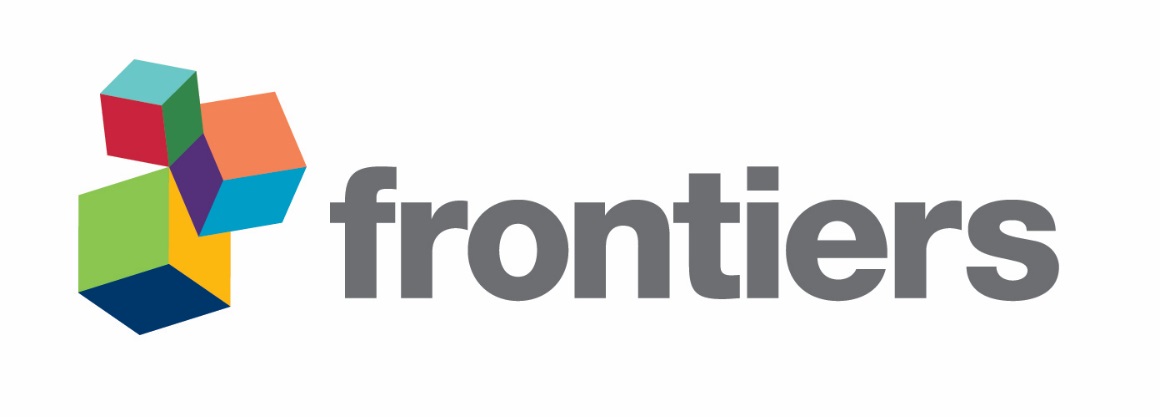
**
